# A lung cancer nurse specialist conceptual model of practice: a mixed methods study

**DOI:** 10.1007/s00520-025-09833-8

**Published:** 2025-08-08

**Authors:** Renae Grundy, Jane O’Brien, Farida Saghafi, Christine Stirling

**Affiliations:** 1 Royal Hobart Hospital, University of Tasmania, Tasmania, Australia; 2 Queensland University of Technology, Brisbane, Australia; 3 University of Tasmania, Tasmania, Australia

**Keywords:** Person-centred care, Lung cancer nurse specialist, Oncology nurse specialist model of practice, Group concept mapping, Carers and people with lung cancer

## Abstract

**Purpose:**

To identify and conceptualise the functions of the Lung Cancer Nurse Specialist (LCNS) role as reported by individuals with lung cancer, their carers, and LCNSs. Presented as a model of practice for the LCNS that strengthens evidence surrounding the LCNS role and optimises meeting the needs of people with lung cancer and their carers.

**Study design:**

A mixed methods approach using Group Concept Mapping (GCM) was employed. GCM is a participatory approach that includes sequential qualitative brainstorming and quantitative analysis using multidimensional scaling and hierarchical cluster analysis. Forty-six participants completed the brainstorming data collection, and 18 completed the sorting and rating data collection. Participants were people with lung cancer, their carers, and practicing LCNSs from Australia.

**Results:**

A conceptual model of practice was developed identifying six key functions: person-centred care, dependable accessibility, individualised information provision, professionalism, specialist nurse, and coordinator. All participant groups (people with lung cancer, carers, LCNSs) rated person-centred care highly, with nuanced differences amongst the other functions. People with lung cancer and carers rated dependable accessibility and individualised information provision highly, while LCNSs prioritised professionalism.

**Conclusion:**

The conceptual model developed highlights the importance of person-centred care, individualised information provision, dependable accessibility, and professionalism. The importance of including carers in person-centred care is emphasised, and the need for specialist lung cancer nurse-specific training and education. This Model of Practice captures the work of LCNSs that aid in improving the outcomes for those impacted by lung cancer.

**Supplementary Information:**

The online version contains supplementary material available at 10.1007/s00520-025-09833-8.

## Objective

Lung Cancer Nurse Specialists (LCNSs) deliver an important role for people with lung cancer and their carers [1]. Their expertise is also important as advances in the diagnosis and treatment of lung cancer are shifting previously poor survival rates and improving prognosis [2, 3]. This study explores what people with lung cancer, their carers, and LCNSs identify and prioritise as essential aspects of the LCNS role in the Australian context. This information informs a LCNS model of practice designed to better meet the needs of people with lung cancer and their carers in the future.

## Background

The LCNS role has developed in Australia from the early 2000 s, along with many other oncology nurse specialist roles as a result of the 2003 ‘Optimising Cancer Care in Australia’ report. The report called for coordinated, patient-focused, multidisciplinary care and support throughout the cancer journey, emphasising the complexity of managing individuals across diverse providers and settings [4]. The role has matured into an advance practice role, that is, LCNSs function at an advanced level by integrating professional leadership, education, research, and system support into their practice. They demonstrate specialised expertise, critical thinking, and complex decision-making while practising autonomously to ensure safe and effective patient care [5].

In Australia, the introduction of LCNSs was unstructured, resulting in an inconsistent role with characteristics that remained undefined beyond standard nursing competencies [6]. Efforts are underway to develop a standardised approach to defining, communicating, and documenting the LCNS role while also informing practice, policy, and resource allocation [7, 8]. This has led to the creation of the Expectations, Standards, and Performance (ESP) Framework, which is currently being implemented [8]. The framework aligns with the domains of advanced nursing practice as outlined by senior officials [8].

The ‘Optimal Care Pathway for People with Lung Cancer’ guide [9] and the ‘Lung Cancer Framework: Principles for Best Practice Lung Cancer Care in Australia’ document [10] recommend every person diagnosed with lung cancer has access to a LCNS. Yet a recent survey covering 72% of lung cancer treatment facilities in Australia reveals varied LCNS availability, with only 46.8% having an LCNS present [11].

The LCNS role is essential in advocating for people with lung cancer, their carers, and the role itself. A strong and consistent body of evidence to enhance the visibility, recognition, validity, and influence of the LCNS role has been called for [12]. This research conceptualises the key attributes of the LCNS role that people with lung cancer and carers identify as important and the aspects of the role they value most.

## Study design and methods

In order to identify and organise ideas related to the role of the LCNS from the perspectives of people with lung cancer, their carers, and practising LCNS, group concept mapping (GCM), a mixed methods participatory research approach, was used. This approach has proven useful in healthcare research for gaining multiple perspectives [13]. More than 300 GCM studies have been published since 2012 in fields ranging from addiction to chronic disease to nutrition and women’s health [14]. The process of GCM uses six steps [13]: preparation—study objectives were defined, and a focus prompt was developed; generation—participants brainstormed ideas in response to the prompt; structuring—participants sorted the unique statements into groups based on perceived similarity and rated each statement on importance using a Likert scale; analysis—quantitative techniques, including multidimensional scaling and hierarchical cluster analysis, were applied to create visual maps; interpretation—the research team engaged in an iterative, discursive process to name and refine the clusters; utilisation—a final conceptual model is developed. As a sequential mixed-methods methodology, the qualitative component occurs when participants brainstorm ideas, some of whom then sort and rate the results. This is followed by quantitative analysis using multidimensional scaling and hierarchical cluster analysis [14]. Sophisticated and rigorous multivariate data analyses is used to construct maps. The series of maps produced visually depict the composite thinking of the participants and result in maps constituting a framework that can be immediately used to guide action [13].

Guidelines suggest that the participant numbers for the brainstorming phase are typically 10–50 [15, 16]. The sorting and rating phase requires a subset of 15–40 participants to be effective for generating meaningful cluster structures [17]. The overall recommendation is 30–60 participants for robust results [14, 16].

### Ethics

This research was granted ethical approval on 25 June 2019 by the Tasmanian Social Sciences Human Research Ethics Committee, approval number H0017980, and was conducted in accordance with the Declaration of Helsinki.

### Participants

The study recruited people with lung cancer, carers, and LCNSs in Australia with participants self-identifying as one of these groups. A ‘carer’ was defined as a partner, family member, friend, or anyone significant to a person with lung cancer. Recruitment was through convenience sampling, and participants had to be over 18 years of age and able to read and understand English. Participation was anonymous and limited to those in Australia. Recruitment used a Facebook page, an online platform, and hard-copy ads. Participants confirmed eligibility and provided consent via a website or requested a paper-based pack that included an information sheet with consent. Consent via paper-based participation was by completing the paper version consent form and returning it in the pre-paid and addressed envelope. Online participants had a consent page; they had to select an ‘accept’ or ‘reject’ button to move forward.

### Preparation and generation

Preparation included the research team formulating and refining the focus prompt statement. The focus prompt for this study was, ‘If the lung cancer nurse specialist role were the best it could be, the lung cancer nurse specialist would….’ Following common GCM practice, the focus prompt used a sentence completion format, guiding participants to brainstorm in a structured manner [18]. Forty-six participants responded to the focus prompt. Responses provided the brainstorming statements to conduct the next steps. Participants could answer the focus prompt as many times as they liked, keeping a single idea for each answer, and providing syntactically similar data [18]. Collecting brainstorming statements ceased after more than 100 statements were achieved [15]. Brainstorming was done online and on paper, both valid ways of data collection [18]. The research team then entered the paper responses into the online software, edited the statements to remove repeated answers and split up statements that contained more than one idea [18]. Through this process, from 159 total statements, 101 unique statements were available for sorting and rating.

### Structuring—sorting and rating

A second recruitment phase for sorting and rating was used to ensure diverse perspectives and enhance methodological rigour. While initial participants generate ideas, recruiting a separate or expanded group for sorting and rating helps to mitigate bias, improve representation, and maintain feasibility [16]. This approach strengthens the validity and reliability of the concept mapping process and mitigates natural attrition that may occur [16]. With further advertisements placed on the Facebook page, an online platform, and hard-copy advertisements, 18 participants were recruited (9 people with lung cancer, 4 carers, and 5 LCNSs) and completed sorting and rating. Of the five LCNS, four were return participants, all other participants were new.

**Fig. 1:**
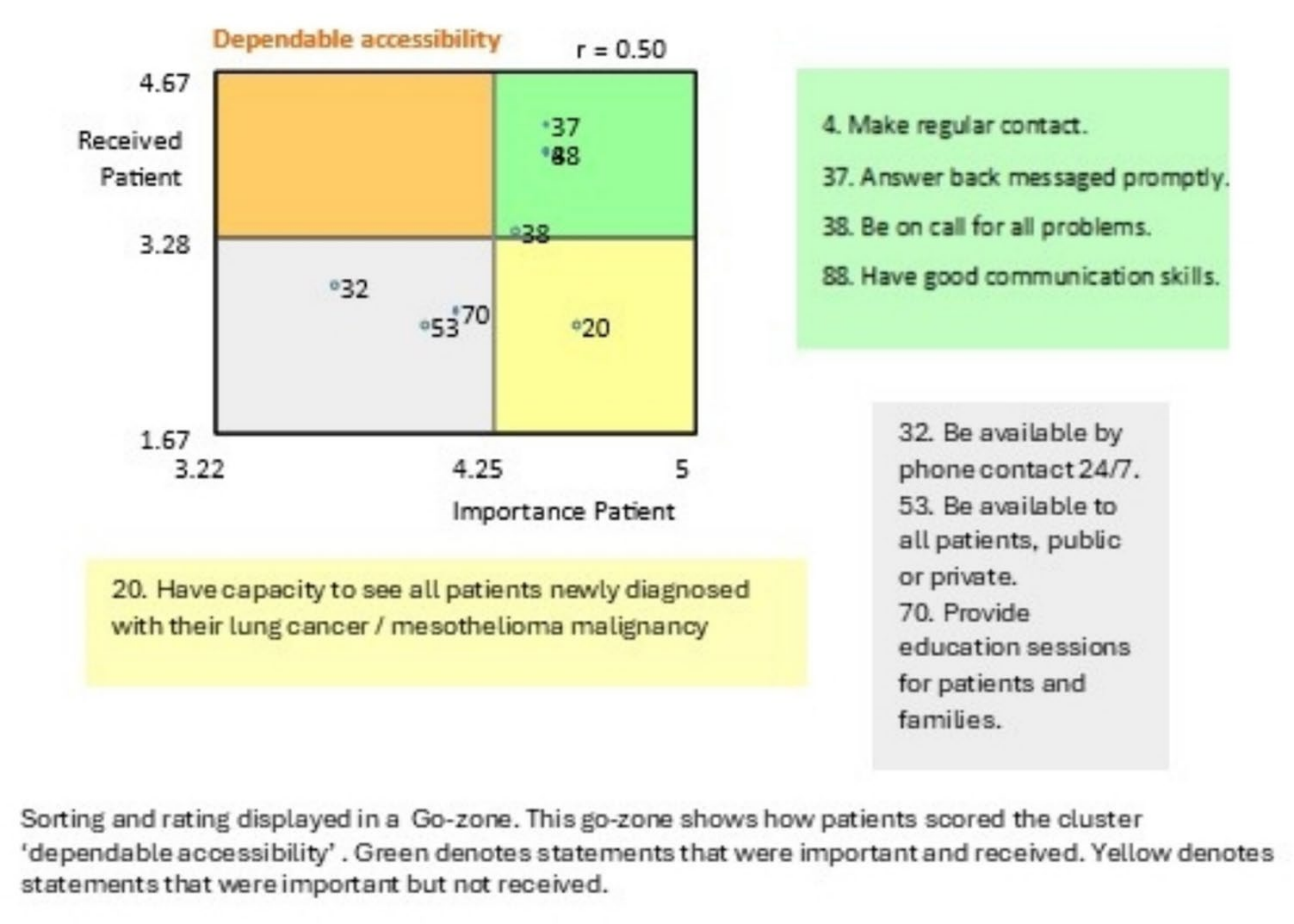
Go-zone example for sorting and rating

During sorting, each participant examined the full set of statements collected in the previous stage and sorted them into homogenous groups based on their perceived similarity [19] in a way that made sense to each person. Individual statements can only be placed in one group, and there must be more than one group [15]. During rating, each participant attached a value to each idea based on two variables using a Likert scale [15]. The Likert scale for this study asked participants to rate the importance of the brainstorming statements and whether they received the care or process described in the brainstorming statement if they were a person with lung cancer or carer, or if they delivered the item if they were a LCNS. See the example provided in Fig. 1.

### Data analysis

**Fig. 2:**
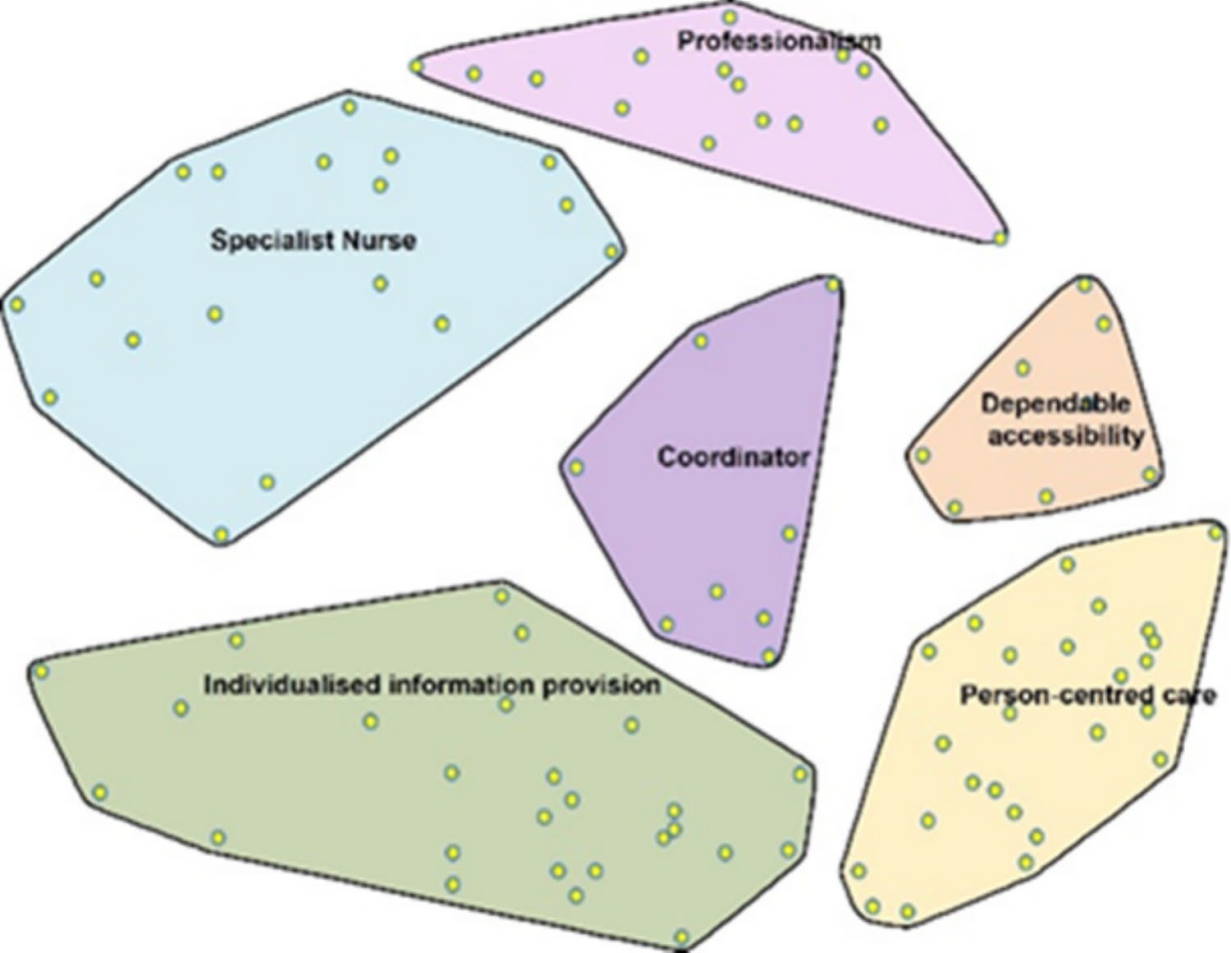
Cluster map

Integration of the qualitative and quantitative data occurred via the software. Data were entered into the CS Global MAX™ software, where qualitative data were converted into quantitative two-dimensional maps. Binary was used to represent the qualitative data as quantitative and represented the ideas of every individual participant in relation to all other ideas [18]. The CS Global MAX™ software then used a multidimensional scaling algorithm to create a point map. Multidimensional scaling is a way to visualise the level of similarity of individual statements in a dataset. This point map is a two-dimensional relational representation of each statement [16]. A cluster map was created from the point map (Fig. 2).

### Interpretation and using the results

After analysing various cluster solutions, the research team selected a six-cluster map as the best representation of the data. Cluster names were derived from participants’ sorting labels and the statements within each cluster. The research team synthesised this information to determine overarching themes, ensuring the final cluster names reflected participant perspectives.

## Results

### Participants

Forty-six participants completed brainstorming, and 18 participants completed sorting and rating; Table 1 describes the split between both phases of recruitment. Of all participants completing both phases of the research, the average age of participants was 59 years (range 20 to 92). Most of the participants were female (*n* = 61.71%). People with lung cancer comprised the majority of participants, with similar numbers of participants being carers and LCNSs. The number of non-respondents to the demographic questions was about a third (*n* = 13, 15%). On average, all participants had been involved with lung cancer for 5 years, and most were close to their place of care, with 51 (59%) less than an hour of travel time from their care centre. No participants were more than 3 h away.

**Table 1:** Participant demographics per phase of study

Brainstorming			Sorting and rating		
People with lung cancer	*N* = 28	60.87%	People with lung cancer	*N* = 9	50%
Carers	*N* = 5	10.87%	Carers	*N* = 4	22.22%
LCNS	*N* = 9	19.57%	LCNS	*N* = 5	27.78%
Did not respond	*N* = 4	8.7%	Did not respond	*N* = 0	0%
Total	*N* = 46	100%	Total	*N* = 18	100%

### Cluster summary

**Fig. 3:**
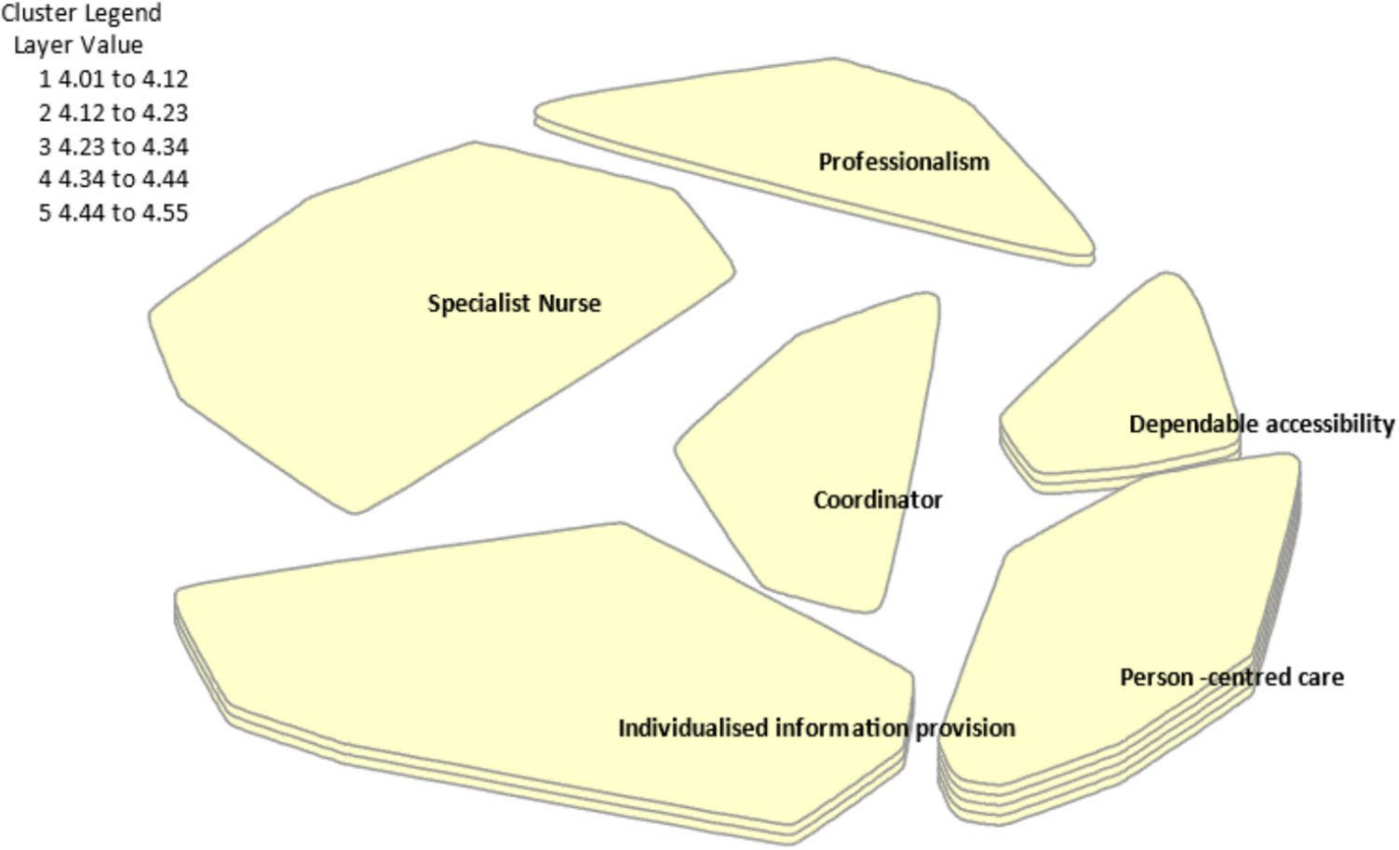
Cluster map by importance for all participants

The six clusters selected from the data were person-centred care, dependable accessibility, individualised information provision, professionalism, specialist nurse, and coordinator. Clusters are presented in order of importance as ranked by all participants. Figure 3 demonstrates this, where the more layers present for each cluster indicate a higher importance ranking for that theme.

The aspects of the LCNS role described by participants were categorised into process activities (actions that structure care delivery) and value activities (actions that enhance experience and satisfaction). The distinction between these categories emerged from participant descriptions, which were analysed to identify common themes. Process Activities include making regular contact with people with lung cancer, meeting people when lung cancer is first suspected, using a checklist to guide people through the lung cancer trajectory, receiving formal training in needs screening, and providing referrals to allied health. Value Activities include explaining medical terminology in an accessible way, enquiring about individuals’ emotions and concerns, encouraging open and honest communication, and being sensitive to cultural diversity.

Underlying the entirety of the LCNS role is professionalism. All elements of the clusters can be seen to be overlapping, with elements of person-centred care occurring in all other clusters for example. Each participant group (people with lung cancer, carers, and LCNS) found similar clusters important. However, there were nuanced distinctions. All groups rated the cluster ‘person-centred care’ as highly important. Both caregivers and people with lung cancer ranked their top three in the same order: ‘person-centred care’, ‘dependable accessibility’, and ‘individualised information provision’. LCNSs prioritised ‘professionalism’ as the most crucial for the role, followed by ‘person-centred care’, ‘individualised information provision’, and ‘specialist nurse’. Both people with lung cancer and carers rated ‘dependable accessibility’ much higher than LCNSs did. Clusters ‘specialist nurse’ and ‘professional collegiality’ clusters were rated higher by LCNS than by people with lung cancer and carers.

Caregivers assigned a higher rating to the ‘coordinator’ cluster compared to people with lung cancer. Carers rated ‘individualised information provision’ and ‘coordinator’ higher than people with lung cancer and nurses. People with lung cancer valued ‘coordinator’ less than LCNSs and carers. LCNSs felt that they delivered ‘dependable accessibility’ and ‘coordinator’ the least, but people with lung cancer and carers felt that this was delivered the most. A table of all 101 statements is available in the supplementary file, arranged by cluster and ratings.

#### Person-centred care

This cluster contains statements expressing the LCNS being with the individual with lung cancer and their carer during the cancer journey. It describes a desire for the LCNS to keep the person at the forefront of the chaos of a cancer diagnosis. This cluster and the ‘individualised information’ cluster contain the most statements, 25 and 26, respectively. Elements of being a skilled communicator and having expert knowledge of the lung cancer pathway are indicated in the brainstorming statements in this group. Examples of statements in this cluster are provided as follows by statement number:

*34. Be attuned to mental health needs of patients while they are trying to cope with life being turned upside down.*

*77. Help and acknowledge family/carers and the important role they can play in supporting the patient.*

*14. Meet me when lung cancer first suspected.*

#### Individualised information provision

This cluster is related to practical concerns and includes the impact of cancer and its treatments on the individuals’ body. It describes desired support or available support, either from the LCNS, allied health, or not-for profit organisations, as well as practical basic information such as contact numbers, any financial costs, hospital maps, and subsidised travel. As the largest cluster by area, it demonstrates the variety of support expected by people:

*1. Have a checklist for each patient and make sure all items are covered off in a timely manner, especially if treatment does not follow usual process, e.g., are all baseline scans done.*

*72. Provide a ‘menu’ of services available to patients as options to help provide holistic care.*

*98. Be sensitive to cultural diversity.*

#### Dependable accessibility

This cluster reveals a desire for the LCNS to be easily accessible and often. It contains eight statements emphasising the need for open and consistent communication channels. The LCNS is a central point of contact for both people with lung cancer, carers, and the wider lung cancer team:

*4. Make regular contact.*

*37. Answer back messages promptly.*

*20. Have capacity to see all patients newly diagnosed with their lung cancer/mesothelioma malignancy.*

#### Professionalism

This cluster outlines the conditions that enable an LCNS to perform the role at its best and includes desirable attributes of the nurse. Factors such as workplace conditions, and networks made with other LCNSs were found in this cluster. The need for educational opportunities and support for development also occurred in this cluster:

*30. Perform role with dignity and professionalism.*

*47. Have a manageable case load.*

*51. Be involved in lung cancer advocacy activities in their community and nationally.*

#### Specialist nurse

This cluster covers the LCNS as a leader in the field of lung cancer; clinically, as an advocate and a spokesperson. The cluster, one of the largest by map area, contains 18 statements, showing the diversity of leadership domains:

*74. Be very knowledgeable about all types of lung cancer and gene mutations, all types of treatments including different types of surgery, types of radiation, chemo, targeted therapies, immunotherapy, and combination therapies.*

*100. Be skilled in symptom management.*

*42. Lead nurse led symptom and self-care clinics.*

#### Coordinator

This cluster is related to practical assistance and ease of movement throughout the cancer journey, as well as a need for a central link with the system to explain and coordinate ‘medical stuff’. The cluster contains nine statements. It is implied in the statements that nurse-led clinics in this cluster would be for a needs assessment, with person with lung cancer and carer present, allowing for ease of referrals to outside services that the person with lung cancer may need:

*43. Have follow-up lung cancer nurse care provided at designated time-points during the patient’s lifetime.*

*46. Have good and easy access to allied health services.*

*89. Liaise with patient’s GP.*

## Discussion

**Fig. 4:**
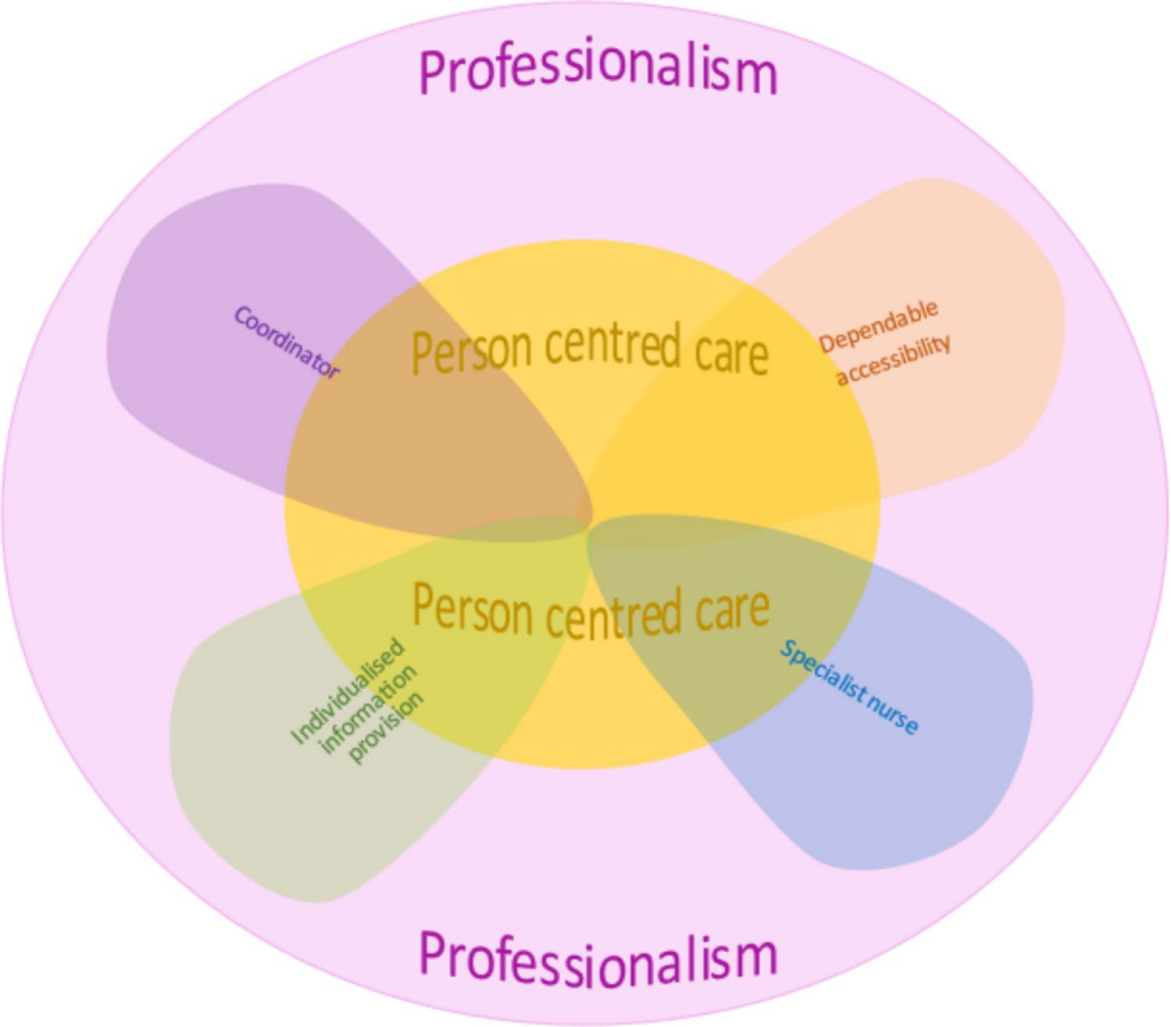
A conceptual model of practice for the LCNS role

Each cluster will be discussed in turn in relation to existing literature, drawing on the statements contained in the cluster to provide discussion points. Figure 4 shows a conceptual model described by this research. From the data, facets of the LCNS role perceived by participants as done well and valued were extracted. By capturing, naming, and describing these facets, the participants conceptually described the LCNS role.

### Person-centred care

Person-centred care (PCC) influences all facets of the LCNS role; hence, it is centrally placed in the conceptual model (Fig. 4). Organisations hold competing demands on nurses for space to carry out PCC, including time, workplace culture, workload, and financial restraints, to name a few [20]. Nurses delivering person-centred care are often impeded by the resources of their work environment, their own competence and communications skills, and policies affecting their practice. Leary and colleagues [21] describe this as the work left undone.

Carers want to be included in person-centred care. Carers expressed they wanted to be recognised for the support they provided to the individual with lung cancer, and their needs do vary from those of the person with lung cancer. Nurses implement person-centred care by recognising the uniqueness of the person with lung cancer, and this in turn extends to their family [20].

Communication skills are essential to PCC, and this facet was present in all clusters. It is noted throughout the literature the LCNS should be a skilled communicator [22]. Empathetic responding to reduce lung cancer stigma is a current trend in communication training being offered to any healthcare worker that has contact with people with lung cancer [23]. People and carers, when dealing with an advanced lung cancer diagnosis, want clear and honest information delivered empathetically. They want the carer included, and they want the key worker, such as an advanced practice nurse, to be present to ensure continuity of communication across all those involved in the person with lung cancer’s care [24].

Nurses experience more job satisfaction after assertiveness training, and, importantly, patient outcomes are improved [25]. As found by Stewart [26] and team, assertiveness and MDT engagement are linked. In the UK, Stewart [26] and team found just over half (51.7%) of the LCNSs in their study would challenge any of the other members of the MDT, and a fifth (19.1%) of the LCNSs found MDTs intimidating or uncomfortable [26]. Carers and LCNSs both felt the LCNS was assertive, and that this was important.

### Individualised information provision

Carers rated receiving individualised information more important than other participants and felt they received it less often. Coder’s [27] research echoed these findings when exploring the experiences of family members of people with terminal cancer. The carer participants in Coder’s [27] study sought further information outside the clinical setting in order to aid their own comprehension, and also acquired information for the patient, and sought further information to educate other family members and to communicate with the treating team [27], implying that their information needs were not being met.

Langbecker [28] and team describe how communication skills and attitudes of health professionals influenced the amount and type of information they provided to patients. In this cluster, ‘information needs surrounding cultural sensitivity’ received high importance and low delivery ratings, and this is reflected in the literature. Culturally and linguistically diverse (CALD) people with lung cancer experience a delayed diagnostic interval in hospital compared with Anglo-Australian people [29]. An Australian study in 2017 found that oncology nurses generally have low confidence when working with minority groups [30]. There is a known lack of training opportunities for CALD oncology communication competency for health professionals [30]. An earlier study [31] suggests that cross-cultural training needs to be included in oncology training for nurses.

### Dependable accessibility

This cluster showed that all participants valued the LCNS making regular contact, having effective communication skills, and replying promptly to messages and that these activities and skills were delivered. Being accessible is key to developing and maintaining a therapeutic relationship, knowing the nurse is available face-to-face or via the phone [32], and providing a flexible service [33]. The LCNS being a central point of contact is a common theme throughout the literature, not only for people with lung cancer and carers but for the whole multi-disciplinary team [22]. McPhillips and colleagues [22] call this role a ‘named key worker’ and indicate the presence of an LCNS improves the overall the individual’s experience of the lung cancer trajectory. The present study reflects similar findings, describing dependable accessibility as providing a flexible service—that is, able to offer real-time responses face-to-face or by phone. This is valued by both people with cancer and physicians [33].

People with lung cancer and carers felt the LCNS did not provide dependable accessibility as they were not able to see all newly diagnosed people with lung cancer. LCNSs and carers felt LCNSs were not available to all people with lung cancer, regardless of private of public health care status. There are many possible reasons for this, including demands of the employing service, workload, LCNS funding, and the position being part-time. In the UK, the target is that a LCNS will see at least 80% of people with lung cancer, and at least 80% of individuals should have an LCNS present at diagnosis [22]. The flexibility of the LCNS role, and being able to respond in real time, can come at the cost of increased workload [33], such as having scheduled appointments and needing to respond to issues arising from phone calls, and competing administration requirements.

### Professionalism

Professionalism in nursing is a multifaceted concept that encompasses the knowledge, attitudes, and behaviours underpinning effective clinical practice. Professionalism entails delivering compassionate, evidence-based care tailored to the complex needs of oncology patients, while upholding ethical standards and demonstrating accountability within the healthcare team [34].

Professional identity is a way to ease role ambiguity and enhance role legitimacy for LCNS. Fitzgerald [35] conducted a concept analysis surrounding professional identity. They found that it can be constructed from the behaviours and activities of the role; the more an individual identifies with these, the greater the professional identity and, in turn, the higher the job satisfaction [35].

Determining a caseload is difficult due to the many variables at play including the type of institution the LCNS works in and the geographical location of their work. Suggested caseload numbers can be found from the UK; for a full-time LCNS, new cases of people with lung cancer should not exceed 80 per year [36, 37]. More work is required in Australia to determine whether case numbers, the geography of health services across rural and remote areas, and the needs of the Indigenous population will have an impact on caseload. At times, the LCNS takes on administrative tasks to the detriment of time spent with patients and carers [22, 38].

LCNS participants in this study listed ‘having dedicated time to conduct nurse-led lung cancer research programs’ as important but not occurring. This is important to further advance both the LCNS role and to improve outcomes for patients and carers. Many nurses feel a lack of confidence about how to conduct research, mentors can be an invaluable resource in this area [39]. Principle 5 of best-practice lung cancer care in Australia from Cancer Australia [40] (2018, p. 33) is ‘data-driven improvements in lung cancer care’. It is echoed in the literature that oncology nurses face barriers to conducting research. Including the availability of paid time to write proposals, for analyses of data, writing up results, lack of skills and knowledge on how to conduct research, and knowing what resources are available to them to support research [41].

### Specialist nurse

This cluster is concerned with the LCNS being an advanced practice nurse, clinically skilled, and knowledgeable. Across all participant groups in the current study, two statements were made about aspects universally considered to be important to the LCNS role and being delivered: ‘be skilled in symptom management’ and ‘be knowledgeable about all types of lung cancer and all types of treatments surrounding lung cancer’. Nurse led follow-up clinics may be a way this facet of the LCNS role can be achieved. They are acceptable to people with lung cancer and have high satisfaction ratings [42]. Overall, clinics in cancer care led by advanced practice nurses are well accepted but there is a broad range of scope and style in their delivery [43]. Molassiotis [43] and colleagues in a scoping review found only a small number of trials (17) investigating nurse-led clinics within oncology over the past 20 years, the majority being focused on breast cancer. One trial, however, was lung cancer-specific; see Moore [44] and colleagues.

Other approaches to nurse led clinics have been explored. Krug [45] and colleagues conducted a study with people with stage four lung cancer and their informal caregivers. This was a milestone communication approach, the milestones were baseline, then 3, 6, and 9 months, and conducted by a nurse/physician in tandem. The nurses in the study group were provided with communication training prior to the study commencing. It is not specified whether the nurses discussed in the study were LCNSs or oncology nurses. Krug [45] and team found patients in the study group had decreased information needs compared to their control group receiving standard oncology care.

### Coordinator

As a way to streamline cancer care, proactive case management style of LCNS nursing has been suggested as a way to mitigate potential problems along the lung cancer trajectory for people with lung cancer and carers. This style of LCNS nursing has led to a decrease in emergency department presentations [46]. A milestone communication approach, that is, situation-specific conversations throughout the lung cancer trajectory, has proven beneficial and decreases information needs [45]. As patients’ needs tend to fluctuate around certain time points, such as at diagnosis, the start of treatment, recurrence of cancer, or moving into a survivorship phase [27], this style of nursing may match.

Participants with lung cancer noted that help with navigating the hospital system was delivered. LCNSs possess intricate working knowledge of the health system, including access to allied health, liaising with GPs, and navigating the hospital system. Wagner [47] and team called this ‘local care system savvy’ (2014 p. 17). This concept of care system savvy is also noted by Tod [48] and colleagues, describing skills in communicating, navigating, and brokering as characteristics of the LCNS role; that is, negotiating for patients across and between services, professionals, and organisations.

## Implications of findings

The research described in this study offers new insights into the views of people with lung cancer and their carers, research with carers of people with lung cancer being extremely limited [1, 49]. Across the three participant groups, there is a clear synergy in what is considered important and essential to the role.

This conceptual model of practice for the LCNS provides a snapshot for ongoing refinement and evidence-building for a practice model for the LCNS role. By articulating these essential aspects of the LCNS role within the Australian context, this model strengthens the evidence base for LCNS practice and supports the delivery of care to promote better outcomes for individuals affected by lung cancer.

## Limitations

Although the number of participants met the recommended range for GCM [15], the number of participants from both people with lung cancer (*N* = 36) and carer (*N* = 9); participant type not answered (*N* = 4) groups may not be adequate to provide a true representation of the experience of the LCNS role Australia-wide, particularly for the carer cohort. Demographic data collected did not ask for specifics regarding remoteness, Indigenous status, nor CALD information. Demographic data did not capture what level or type of education and training the LCNSs in this study had received. This may have proven useful in making future recommendations. However, many of them were based in large metropolitan centres, again, not giving satisfactory representation of rural and remote areas.

The generalisability of the findings may be limited as only the Australian context is reflected. For individuals with lung cancer, the stage of their lung cancer was not captured, such as stage 1 and curable, or end stage and palliative. Thus, the generalisability of the concepts may not be applicable in all circumstances.

## Conclusion

The Conceptual Model of Practice for the LCNS role produced in this study provides a framework for ongoing research and the continuing building and defining of a model of practice. This conceptual model aids in describing what people with lung cancer, carers, and LCNSs see as the key activities and values that make up the role of LCNS. This research has added to the ongoing development of evidence validating the LCNS role. The produced Conceptual Model of Practice for the LCNS role informs the practice of the LCNS as it is now and may inform how to progress it to improve the lung cancer care experience for people with lung cancer and their carers.

## Supplementary Information

Below is the link to the electronic supplementary material.

Supplementary file 1 (DOCX 26.9 KB)  

## Data Availability

No datasets were generated or analysed during the current study.
