# Supplementary material for: A lung cancer nurse specialist conceptual model of practice: a mixed methods study
Source: Support Care Cancer. 2025 Aug 8;33(9):766. doi: 10.1007/s00520-025-09833-8 (PMC12334486; doi:10.1007/s00520-025-09833-8)
Supplement: Supplementary file 1 — (DOCX 26.9 KB) [file 520_2025_9833_MOESM1_ESM.docx]

A Lung Cancer Nurse Specialist Conceptual Model of Practice: A Mixed Methods Study

**Supplementary file:** Brainstorming statements organised by cluster. The importance of the statement and if the item was received by a patient with lung cancer, or their carer or delivered by a LCNS is also shown.

| **Cluster** |  |  | **Importance** | **Rec/Del** |
| --- | --- | --- | --- | --- |
| **Coordinator** |  |  | 4.01 | 2.88 |
|  | 11 | Explain medical jargon in understandable terms. | 4.44 | 3.61 |
|  | 43 | Have follow-up lung cancer nurse care provided at designated time-points during the patient’s lifetime. | 4.39 | 3.22 |
|  | 46 | Have good and easy access to allied health services. | 4.28 | 3.06 |
|  | 89 | Liaise with patient's GP. | 4.22 | 3 |
|  | 10 | Help navigate the hospital system. | 4 | 3 |
|  | 19 | Ensure we have retained medical information correctly by giving us a follow up phone call after appointments. | 3.89 | 2.72 |
|  | 16 | Make sure I receive the doctors’ requests for pathology tests etc. | 3.72 | 2.61 |
|  | 17 | Assist in coordinating appointments. | 3.72 | 2.33 |
|  | 18 | Assist in coordinating hospital appointments on the same day. | 3.44 | 2.33 |
| **Individualised information provision** |  |  | 4.27 | 3.04 |
|  | 2 | Provide a list of things the nurse can be contacted for. | 4.83 | 4.72 |
|  | 6 | Provide information on what the lung cancer nurses role is. | 4.78 | 4.11 |
|  | 83 | Provide adequate printed information for the patient and their family to enable them to better understand lung cancer and treatments. | 4.78 | 4 |
|  | 3 | Provide contact number. | 4.78 | 3.67 |
|  | 24 | Clarify what I can expect from treatment I am receiving. | 4.67 | 3.67 |
|  | 78 | Where there are multiple treatment options, explain the detail of each so patients understand enough to help them in making a decision, including likely side effects and effect on quality of life. | 4.67 | 3.61 |
|  | 82 | Ensure every patient is given contact details for who to contact if they have questions or concerns between appointments. | 4.61 | 3.56 |
|  | 23 | Clarify symptoms I can expect from treatment. | 4.61 | 3.56 |
|  | 5 | Provide information on other services, e.g., Cancer council, Lung Foundation, palliative care, respite care, internet support groups. | 4.61 | 3.5 |
|  | 73 | Help patients manage all types of treatment related side effects / toxicities (surgical, radiation chemo); physical, emotional and psychological, by being a contact point, providing support and strategies. | 4.56 | 3.39 |
|  | 98 | Be sensitive to cultural diversity. | 4.44 | 3.39 |
|  | 85 | Explain exactly what will come next and when, e.g., what appointments for what purpose, where, how long for results etc. | 4.44 | 3.33 |
|  | 22 | Provide referrals to allied health such as dietitian, social work, Occupational Therapy, physiotherapy, smoking cessation nurse and psychologists when needed. | 4.39 | 3.28 |
|  | 72 | provide a 'menu' of services available to patients as options to help provide holistic care. | 4.39 | 3 |
|  | 1 | Have a checklist for each patient and make sure all items are covered off in a timely manner, especially if treatment does not follow usual process, e.g., are all baseline scans done. | 4.39 | 3 |
|  | 31 | Update me on any new information relating to treatment. | 4.28 | 2.94 |
|  | 7 | Provide a summary of what has happened up to this point. | 4.22 | 2.94 |
|  | 36 | More explanations on what cancer is, how it happened and why things cannot be done. | 4.22 | 2.72 |
|  | 33 | Alert me to any costs associated with treatment earlier rather than later. | 4.17 | 2.56 |
|  | 91 | Would come to appointments with people from cultural backgrounds where Cancer is considered as punishment for something bad you did in a former life. | 4.11 | 2.56 |
|  | 12 | Assess patients as to when a phone consultation or face to face consultation is required, to save patients a trip to the hospital. | 3.78 | 2.44 |
|  | 65 | Provide me with a map of the hospital. | 3.67 | 2.11 |
|  | 21 | Inform patient subsidised travel is available. | 3.56 | 1.89 |
|  | 90 | Would organise a group of buddies or volunteers where people with limited English, poor literacy and no IT, or alone, or vulnerable, can be paired with an Australian person who would help them navigate the system. | 3.39 | 1.78 |
|  | 68 | Would take me to see where treatments will happen. | 3.28 | 1.72 |
|  | 92 | Provide interpreters. | 3.28 | 1.5 |
| **Dependable accessibility** |  |  | 4.24 | 3 |
|  | 20 | Have capacity to see all patients newly diagnosed with their lung cancer / mesothelioma malignancy | 4.72 | 3.94 |
|  | 88 | Have good communication skills. | 4.61 | 3.94 |
|  | 4 | Make regular contact. | 4.61 | 3.72 |
|  | 37 | Answer back messaged promptly. | 4.5 | 2.72 |
|  | 70 | Provide education sessions for patients and families. | 4.39 | 2.56 |
|  | 53 | Be available to all patients, public or private. | 4.28 | 2.5 |
|  | 38 | Be on call for all problems. | 3.78 | 2.33 |
|  | 32 | Be available by phone contact 24/7. | 3 | 2.28 |
| **Person-centred care** |  |  | 4.55 | 3.53 |
|  | 59 | Be a point of contact throughout the lung cancer journey. | 5 | 4.44 |
|  | 76 | Be caring and honest. | 4.83 | 4.33 |
|  | 13 | Meet me at first visit | 4.83 | 4.11 |
|  | 14 | Meet me when lung cancer first suspected. | 4.78 | 4.06 |
|  | 58 | Being made aware they are available by phone for support, questions or any problems. | 4.78 | 3.89 |
|  | 71 | Ask patients and carers what matters most to them. | 4.72 | 3.83 |
|  | 27 | Encourage open and honest communication, so patients do not put on a brave face. | 4.72 | 3.72 |
|  | 28 | Make it clear at start there is extra help available for support. | 4.67 | 3.72 |
|  | 25 | Enquire how I'm feeling about things. | 4.67 | 3.61 |
|  | 60 | Meet patient and family at diagnosis. | 4.67 | 3.61 |
|  | 75 | Answer any questions to the best of their knowledge. | 4.61 | 3.61 |
|  | 77 | Help and acknowledge family / carers and the important role they can play in supporting the patient. | 4.61 | 3.5 |
|  | 81 | Get to know the patient as a person, because we patients are much more than just a disease on legs. | 4.61 | 3.5 |
|  | 34 | Be attuned to mental health needs of patients while they are trying to cope with life being turned upside down. | 4.61 | 3.5 |
|  | 29 | Gauge mental toll on patients and make appropriate referrals for support. | 4.61 | 3.44 |
|  | 26 | Inform of us of next step and what to expect to help relieve anxiety of not knowing what is going on. | 4.56 | 3.33 |
|  | 64 | Provide support for family / carers. | 4.56 | 3.22 |
|  | 15 | Be with patient from beginning to end of disease. | 4.5 | 3.22 |
|  | 35 | Provide more communication so patients do not feel forgotten. | 4.44 | 3.22 |
|  | 101 | Be a companion on the journey. | 4.33 | 3.22 |
|  | 39 | Answer all my questions. | 4.22 | 3.17 |
|  | 8 | Ask patients to think about what they want / expect in the future. | 4.22 | 3.17 |
|  | 69 | be able to speak with me about any problem. | 4.22 | 3.17 |
|  | 67 | Provide guidance through the unknown. | 4.17 | 3.06 |
|  | 66 | Come to appointments with me. | 3.83 | 2.67 |
| **Specialist Nurse** |  |  | 4.02 | 2.54 |
|  | 74 | Be very knowledgeable about all types of lung cancer and gene mutations, all types of treatments including different types of surgery, types of radiation, chemo, targeted therapies, immunotherapy and combination therapies. | 4.67 | 4.11 |
|  | 100 | Be skilled in symptom management. | 4.5 | 3.89 |
|  | 84 | Have formal training in screening and psychosocial care. | 4.44 | 3.72 |
|  | 97 | Receive ongoing, up-to-date training on treatments. | 4.39 | 3 |
|  | 9 | Contribute to a comprehensive multi-disciplinary team meeting | 4.28 | 3 |
|  | 57 | Share knowledge and educate all colleagues about lung cancer. | 4.22 | 2.83 |
|  | 93 | Have extensive knowledge on clinical trials and research and assist in getting patients into them. | 4.22 | 2.61 |
|  | 63 | Be involved in regular clinical supervision. | 4.06 | 2.39 |
|  | 99 | Be linked to a research hospital. | 4 | 2.39 |
|  | 40 | Have dedicated time protected to run nurse led lung cancer research programs. | 4 | 2.22 |
|  | 42 | Lead nurse led symptom and self-care clinics. | 3.94 | 2.22 |
|  | 41 | Take nurse led lung cancer care out to the community in rural and remote areas. | 3.89 | 2.17 |
|  | 95 | Would attend support group meetings. | 3.89 | 2.17 |
|  | 86 | Be assertive. | 3.83 | 2.11 |
|  | 80 | Be different for each type of cancers. | 3.61 | 1.94 |
|  | 55 | Would work full time hours in a cancer centre. | 3.56 | 1.72 |
|  | 79 | Be a spokesperson for media. | 3.5 | 1.61 |
|  | 96 | Coordinate online support groups. | 3.33 | 1.56 |
| **Professionalism** |  |  | 4.14 | 2.47 |
|  | 30 | Perform role with dignity and professionalism. | 4.61 | 4 |
|  | 49 | Have the position backfilled when on leave. | 4.56 | 3.22 |
|  | 47 | Have a manageable case load | 4.5 | 2.83 |
|  | 56 | Would have post graduate qualifications in lung cancer nursing. | 4.33 | 2.78 |
|  | 54 | Be part of a comprehensive cancer nursing team. | 4.33 | 2.72 |
|  | 61 | Have strong colleague networks. | 4.22 | 2.61 |
|  | 48 | Have education leave paid for. | 4.22 | 2.33 |
|  | 94 | Have training in grief counselling. | 4.17 | 2.28 |
|  | 51 | Be involved in lung cancer advocacy activities in their community and nationally. | 4.11 | 2.28 |
|  | 50 | Be well supported and respected by management, admin and medical / nursing staff. | 4 | 2.22 |
|  | 52 | Be actively involved in a lung cancer nurse specialist organisation. | 4 | 2.22 |
|  | 87 | Liaise with other lung cancer nurses. | 3.89 | 2.11 |
|  | 62 | Have mentors and mentorees. | 3.83 | 2.06 |
|  | 45 | Be financially rewarded well. | 3.72 | 1.83 |
|  | 44 | Work 8 hours a day maximum. | 3.61 | 1.56 |
